# Comparison of Perceived Dietary Intakes Among International, Multicultural, and Non-Hispanic White College Students

**DOI:** 10.3390/nu18142331

**Published:** 2026-07-16

**Authors:** Donna M. Winham, Abigail A. Glick, Mack C. Shelley

**Affiliations:** 1 Department of Food Science & Human Nutrition, Iowa State University, Ames, IA 50011, USA; aglick03@gmail.com; 2 Departments of Political Science and Statistics, Iowa State University, Ames, IA 50011, USA; mshelley@iastate.edu

**Keywords:** young adults, dietary fat, high fat diet, food preferences, nutrient rich, acculturation, international students, Asian Indian, East Asian, Hispanic

## Abstract

**Background/Objectives**: College life shapes the dietary habits and food preferences of young adults by exposure to new foods and independence in dietary choices. This study described and compared self-reported food intakes and perceived dietary changes among International (34%), Multicultural (28%), and non-Hispanic White (38%) students at a Midwest United States university (*n* = 823; aged 18–34 years; 56% women). Importantly, the analysis extended to distinctions among multiple ethnic subcultures by nativity in the same setting. **Methods:** Students completed an online survey comprising a dietary fat food frequency questionnaire (FFQ), an acculturation scale, and reported intake patterns for alcohol, caffeinated beverages, four nutrient-rich food groups, and perceptions of cultural foods access and diet change. **Results:** International student diets had lower percentages of calories from fat (*p* = 0.006) and they reported less consumption of red meats and hyperpalatable foods compared to US non-Hispanic White and Multicultural students (both *p* < 0.001). Yet, International students showed the largest increases in intakes of these foods (*p* < 0.001). Alcohol consumption rose most sharply among US non-Hispanic White students. Multicultural ethnic subgroups had few significant differences. The Asian Indian, East Asian, and Middle Eastern International students showed strong differences in dietary patterns. Acculturation scales were not consistent predictors of food intake for International students. Middle Eastern and East Asian subgroups had the lowest dominant society immersion scores compared to other International students. **Conclusions:** These findings support the need for disaggregated data by cultural subgroups beyond the ‘international’ classification. Cultural affinity and acculturation have nuanced effects on perceived dietary shifts, underscoring the need for targeted nutrition support for all college students, especially vulnerable International students with limited cultural food access.

## 1. Introduction

Early adulthood is a formative period for the development of future food preferences and dietary patterns. For college students, campus life may be their first opportunity to exercise independence in their diet and food choices [1]. Campus dining offerings can introduce students to a variety of items and broaden taste experiences, including vegetarian options and ethnic cuisines [2]. Institutional-level, community-level, and policy-level factors such as limited food access, grocery costs, and employment obligations may have the greatest influence on individual food choice [3]. However, intrapersonal factors such as unfamiliarity with meal preparation, time conflicts, and studying can further overshadow concerns about food and nutrition [2]. Negative behaviors like reliance on high-calorie snacks, irregular meals, and low intakes of nutrient-rich foods can reinforce unhealthy food preferences, contribute to weight gain, and increase chronic disease risk [1,4].

The US has historically been the top destination for International students pursuing higher education. In 2024, over 1.1 million, or 6% of all US college students, were International, with the largest groups from India (29%) and China (25%) [5]. International students may face challenges in finding cultural foods or ones that meet their preferences for taste, freshness, and type. Even when available, the purchase price and/or cost of transportation to stores may be prohibitive [6]. Employment and financial aid may be restricted for International students in the US. Thus, International students may opt for less-desired or less healthy food items that are convenient, inexpensive, and available, e.g., pre-packaged and fast foods [6,7]. In essence, cultural food limitations and financial constraints can lead to forced dietary acculturation and food insecurity [7,8]. In some studies, International students expressed dissatisfaction with US food options, felt they ate healthier in their home country, and wanted to learn to cook their ancestral foods [9,10]. Shifting to learning new cooking techniques with the substitution of cultural foods can make meal preparation more difficult [11,12]. Those who live near cultural peers may share the commensality of meals and resources to reduce isolation [8]. For students living alone, having the time to procure and prepare native foods may be impractical. The loss of cultural food connections increases psychological distress, disordered eating, and feelings of anomie, or social disconnection, for these sojourners [9,13].

Acculturation refers to the progressive adaptation to the social rules and cultural practices of the dominant culture [8,10]. The degree of change is unique to each individual. Acculturation level can suggest how dietary intakes and preferences shift or blend with the majority food culture [13]. The dynamic, multidimensional, and complex change is dependent upon factors ranging from income, the presence of other cultural group members [14], and the ongoing global nutrition transition [15,16].

Mainstream US diet patterns are often higher in meats, fat, and sugar, and lower in vegetables and fruits [1,4,14]. American cultural norms emphasize more meat consumption compared to other countries, which may introduce conflicts with the traditional dietary patterns of many International students [15]. Avoiding prohibited meats, e.g., pork, or meat in general, can be complex for students who follow religious practices such as Judaism, Islam, or Hinduism. Navigating local cultural mores can be challenging [9,12,14]. In Iowa, where the study university is located, pork production in Iowa is an economic mainstay [17]. University events often feature pork and pork products, further complicating food choices for some students.

Nutrient-rich food options such as pulses (beans, peas, lentils, chickpeas) are staple foods in Asian Indian, Middle Eastern, and European cultures, but not commonly consumed in the US. Fruits, berries, and vegetables are present year-round, but are more cost-prohibitive than in many International countries [18].

Certain food and beverage intakes are known markers of diet pattern change at college [4,6,7,11,19,20]. Notably, all college students may increase their intake of high-fat foods, sugary snacks, caffeinated beverages, and alcohol [1,4,11,19,20]. ‘Hyper-palatable’ foods, or those characterized by high fat, sodium, and/or sugar, are convenient, inexpensive, and readily available as processed snack foods, desserts, or sweets [16,21]. While students may recognize these foods as less healthy, they may be unconcerned about their immediate health because of their youth and the transitory nature of college life [11]. Starchy foods such as potatoes, rice, pasta, and breads are satiating and less expensive, but can be calorie-dense [8,10]. Other studies have shown that college students increase intakes of coffee, tea, and energy drinks [19]. High levels of caffeinated beverages function as stimulants for long hours of studying and work. However, caffeine can interfere with learning by disturbing sleep patterns and pose health risks to college students [19]. Alcoholic beverage consumption and a culture of drinking are common on many US campuses. Nationally, 45% of college students aged 19–20 years old reported drinking alcohol [20]. While these trends are well-documented among US students, there is little quantitative data on the extent of these dietary shifts among International cultural subgroups.

Several studies have assessed dietary acculturation among International students in US colleges [6,7,8,9,10,12,13,14,15]. Qualitative research has identified concerns, preferences, and barriers to guide the formulation of structured hypotheses and surveys. Shi et al. note that to date, most research on dietary acculturation has been conducted with small sample sizes (<100), reported as aggregate “international student” data, or focused on students from one country without contrast to peers [8]. Noyongoyo’s comparison of dietary changes by continental grouping of African and Asian students in the US is a rare exception of disaggregation beyond ‘international’ [15]. Few studies have used formal measures of acculturation or dietary assessment with adequate sample sizes [8]. An acculturation instrument suitable for multiple ethnicities is necessary for cross-comparisons [22]. The acculturation tool needs to account for the multidimensional process of retaining one’s culture of origin and adopting practices of the host society [22,23]. In turn, the use of validated food frequency questionnaires (FFQs) that assess dietary trends has been limited.

This research compares food consumption patterns, including meats, hyper-palatable and high-carbohydrate items, caffeinated beverages, alcohol, and nutrient-rich options of fruits and berries, vegetables, and pulses among three cohorts: International, US-born Multicultural, and US-born non-Hispanic White students. The analysis compares dietary patterns among racial and ethnic subgroups within the International and Multicultural cohorts to better capture cultural heterogeneity often neglected in aggregate analyses. The primary objectives were to: (1) describe demographics, meal sources, and dietary intakes, particularly on fat-related food behaviors; (2) evaluate perceived changes in selected foods since coming to college. Secondary objectives were to: (3) compare perceived food consumption changes by cultures within groups; and (4) describe dietary differences and acculturation scale dimensions between cultural subgroups within the International and Multicultural cohorts. The results show differences in high-fat and high-carbohydrate foods, alcohol, and red meat between the three cohorts, and give previously overlooked evidence of differences by cultural subgroups within the Multicultural and International cohorts in the same setting. Survey data on consumption patterns and attitudes toward plant-based meat alternatives, and food security risk factors are reported elsewhere [24,25,26].

## 2. Materials and Methods

### 2.1. Study Design and Recruitment

The study design was cross-sectional with targeted recruitment via email to all International (*n* = 2387) and Multicultural students (*n* = 4464) as identified by the university’s Office of the Registrar. A random sample of 65% of other student emails (*n* = 28,211) was identified using IBM SPSS Statistics software (Version 26, IBM, Armonk, NY, USA). The Registrar provided emails only for students who had permitted information release and were 18+ years old [27]. All International and US Multicultural students were sent an email invitation in early April 2022 and a reminder email one week later. The randomly selected group did not receive a reminder. To reduce variability in food access, participants who did not live in the same city as the university were ineligible. Since the broad definition of young adulthood is ages 18–34 years, the analysis excluded older participants [28]. Those who completed 75% of the survey and gave an email address received a 5 USD e-gift card. The survey consent statement explicitly stated that completion of the survey was informed consent. The Institutional Review Board of the Office of Research Ethics at Iowa State University (#16-289) approved the research study.

### 2.2. Survey Development

The online survey was drawn from our previous college health and acculturation work [6,7,29,30]. The reference frame for dietary intakes and situational questions was the previous 6 months. Demographics (gender, age, academic year, and marital status), on- or off-campus residence, and university meal usage were collected [31]. Self-reported health was ranked on a 5-point scale (poor = 1, excellent = 5) [32]. Students provided details on the primary food buyer, access to facilities for cooking, and meal preparation self-confidence [33]. Seven food characteristics (taste, price, convenience, nutrition, familiarity, healthfulness, environmental sustainability) in choosing foods were classified on a 5-point scale (not at all important = 1; extremely important = 5) [34]. Students were asked the frequency of difficulty in locating cultural foods and foods for religious practices (1 = very often, 5 = never) [35].

Meal frequencies eaten at home, at restaurants or take-out, or fast-food, were reported (never, 1–3 days per month, 1–2 times per week, 3–4 days per week, almost every day) [36]. Students provided information on the regularity of eating a nighttime meal with someone else (1 = rarely; 5 = always) [36]. Perceived change in diet since coming to college, if they considered themselves religious, and whether or not they ate the same foods most days, were reported on a 5-point Likert scale (1 = strongly disagree to 5 = strongly agree) [37].

Respondents completed the validated 17-item dietary fat FFQ screener developed by Block and associates [38]. The researchers manually entered the reported frequencies into the proprietary NutritionQuest software (https://www.nutritionquest.com/free-tools-fat.html (accessed on 13 July 2026); Berkeley, CA, USA) to assess the percent of calories from fat in the diet based on age and sex [39]. In addition to the dietary fat FFQ, students were asked about their perceived consumption of eight food categories considered markers of diet change since entering college in the published literature [6,8,11,14,15,18,40]. The items were meats, hyper-palatable foods, alcohol, caffeinated beverages, energy drinks, and three groups of nutrient-rich healthy foods (fruits/berries, vegetables, and pulses). Following Wakimoto [41] and Briefel [42], the individual FFQ and other food item intakes were coded as ordinal variables: 1 for once per month or less, 2 for 2–3 times per month, 3 for 1–2 times per week, 4 for 3–4 times per week, and 5 for 5 times or more per week.

Students responded whether their consumption of a food item had decreased, stayed the same, or increased since coming to college, or if they did not eat that item [40,43]. International students answered several questions in addition to those described above. They reported how often they cooked or ate native foods each week, and if so, at which meal settings they ate native foods. They provided their perception if their diet intakes were shifting to American patterns, and whether they felt native or US foods were healthier for them to eat [6,7,11,30]. They gave information on the number of years they had been in the US since birth (range 0.5–34 years) [6].

During study development, 10 International students gave informal interviews for content validity about their perceptions and views on food availability and dietary change in the US [24]. Their opinions supported the six culturally important foods based on the previously validated literature for an adapted food frequency questionnaire (rice, noodles, pulses, soups or stews, flatbreads, and soybeans) [6,7,15,18]. Additionally, prior to data collection, two focus groups of undergraduate students reviewed the survey for assessment of face validity. Four International students, two Multicultural students, five non-Hispanic White students, and three faculty members pilot-tested the survey. Minor adjustments in wording and skip patterns were made before final release.

### 2.3. Acculturation Assessment

The 32-question Stephenson Multigroup Acculturation Scale (SMAS) measured the degree of acculturation. It considers the continuum of adaptation and cultural shift with retention of one’s own cultural values and fitting in with the dominant society [22,44]. It draws on concepts from two widely used acculturation scales for Hispanics—the Bidimensional Acculturation Scale (BAS) [23] and the Acculturation Rating Scale for Mexican Americans (ARSMA) [44]. The use of media and social interactions characterizes cultural affinity in multiple dimensions [22]. The instrument gives a score for ethnic society immersion (ESI) and dominant society immersion (DSI). All International students were routed to the SMAS in the survey. Since cultural influences are not limited to International students, if the Multicultural and White students stated their background influenced their food choices, they also completed the SMAS.

### 2.4. Birth Place (Nativity) and Ethnicity Classifications

As described in Section 2.1, the nativity–ethnicity groupings of International and US-born Multicultural and US-born non-Hispanic White students were primarily from the university classifications. Multicultural students are defined as African American, Hispanic/Latino, American Indian/Alaskan Native, Asian American, and/or Multiracial by the university [45]. Hereinafter, “White” refers to US nationals who are not Hispanic and did not report a Multicultural or recent immigrant background. All participants answered a generation-level question drawn from the ARSMA instrument [44]. Second through 5th+ generation were prompted to choose up to three of 11 listed heritages common in Iowa, e.g., White, Hispanic, African American, German, Irish, or Vietnamese [45]. Their first language learned as a child was selected from six choices (English, Spanish, Mandarin, Hindi, Persian, Arabic).

International students were instructed to select 1st generation status even though they were sojourners, not immigrants. Their list of 12 countries of origin was from the university enrollment data [46]. Up to three cultural backgrounds could be selected. Seven options for native language, or first language learned after birth, or other were listed (English, Spanish, Mandarin or other Chinese dialect, Hindi, Persian, Arabic, Korean) [45]. “Write-in” options for ethnicity or language were available for all respondents.

Eight aggregated cultural groups were based on self-report, cultural similarities, and sample size. These were: (1) White (US Whites, Canadians, Europeans); (2) Asian Indians (including Nepalis); (3) East Asians (Chinese, Koreans, Malaysians, Japanese); (4) Middle Eastern–North Africans including Muslim Pakistanis and Bangladeshis (MENA+); (5) Latin Americans (Hispanics, Caribbean Islanders, Brazilians); (6) Africans and non-Hispanic Blacks; (7) Southeast Asians (Cambodians, Laotians, Vietnamese, Laotians); (8) Native Americans and “others” [18,45,46].

### 2.5. Data Transformations and Analysis

The online data were downloaded in SPSS format from SurveyMonkey (https://www.surveymonkey.com, accessed on 7 April 2022; Momentive Inc., San Mateo, CA, USA). Question responses were examined for completeness, plausibility, and distribution normality. Correlations between dietary intakes, demographics, and acculturation variables were examined. Chi-square or analysis of variance (ANOVA) was utilized to compare variables by the three nativity–ethnicity cohorts, and by ethnic subgroups within the International and Multicultural students. Pairwise differences were assessed using adjusted standardized residuals from chi-square tests with Bonferroni correction applied for multiple comparisons [47]. Statistical results with *p* values < 0.05 were considered significant.

The dietary fat FFQ data were entered into NutritionQuest’s online software [38,39]. The program generates four age- and sex-specific categorical estimates of the percent of calories from fat: less than 30%, 30–35% average, 36–40% high, 40–50% very high [38,39]. Next, the 17 individual food item frequencies were entered into exploratory principal components analysis. Four factors with eigenvalues greater than 1 emerged after varimax rotation. The first factor, the ‘red meat and by-products’ cluster, consisted of ground meat, steaks and roasts, bacon or sausage, cold cuts, and hot dogs or Polish sausages (eigenvalue 3.755; 22% of variance). The second factor comprised five hyper-palatable foods that were high-fat and/or high-carbohydrate (French fries, ice cream, doughnuts and pastries, pizza, snack chips; eigenvalue 1.713; 10% of variance). The third (cheese and dairy, and butter on foods) and fourth (eggs, whole milk, butter in cooking) factors had eigenvalues of 1.4 and 1.2, respectively. Internal consistency for the ‘red meat’ scale was in the ‘acceptable’ classification range for both Cronbach’s adjusted alpha (0.740) and McDonald’s omega (0.746) [47,48]. The hyper-palatable food scale was slightly lower in the high ‘marginally acceptable’ range for internal consistency in exploratory research (Cronbach’s adjusted 0.689; McDonald’s omega 0.683) [48]. Scales for factors three and four had poor internal consistency and were not operationalized. The red meat and hyper-palatable foods frequency summary scales were normally distributed.

The self-reported health options of ‘poor’ and ‘fair’ were combined due to few reports of ‘poor.’ The frequency distribution for years living in the US for International students was right-skewed. Based on the cumulative percentages, it was recoded into five equal categories. The food frequency option of 5 times per week was condensed to 3–4 times per week for chi-square analysis, but not ANOVA.

The SMAS acculturation scale construction was examined for internal consistency through reliability analysis [22]. The 17 variables for the ethnic society immersion (ESI) acculturation scale had a Cronbach’s adjusted alpha value of 0.861, or “good” internal consistency [46]. For the 15-item dominant society immersion (DSI) scale, the initial Cronbach’s alpha value of 0.800 improved to 0.837 after removing the English fluency statement. Since university admissions policy requires English fluency for International students, there was little variation in response to that scale item. Summation of the SMAS responses created continuous scores ranging from 16 to 64 for the ESI, and from 14 to 56 for the DSI. Both scales were normally distributed.

General linear models (GLM) were estimated using the meat and hyper-palatable food scales based on the dietary fat FFQ food items for all cases, and separately for the International and Multicultural students. The initial models included binomial measures for gender, undergraduate or graduate status, marital status, campus dining plan, off- or on-campus residence, native English speaker, International and Multicultural classification, and eight ethnic groupings. Age, perceived health status, diet change, shared meal times, and lack of availability of cultural or religious preference foods were continuous variables. Finalization of the regression models was by sequentially excluding variables with significance levels greater than 0.05 except when retention of the variable improved the model fit [46]. A similar process estimated the ESI and DSI components of the SMAS acculturation scale and the OLR to predict low-fat intake categories.

## 3. Results: International, Multicultural and White Students

### 3.1. Sample Size and Demographic Characteristics by Nativity–Ethnicity

Of the 1158 individuals who started the survey, 35 were not within the 18–34-year age limit, and 68 lived outside of the campus area and were ineligible. The data were examined for credibility, honesty, and completeness. Cases were excluded for failing honesty questions (76) or missing variables needed for analysis (126). The analysis sample of 823 students primarily lived off-campus (72%), were undergraduates (63%), and most of whom were women (56%), with a mean age of 22.7 ± 3.9 years. The sample approximated the university campus demographics for spring 2022 (living off-campus 51%, undergraduates 83%, and women 45%) [45]. In total, 38% percent of the sample were White, 28% Multicultural, and 34% International students.

The three nativity–ethnicity cohorts (International, Multicultural, White) showed significant differences for age, gender, self-reported health, academic level, native English speaker, generation, religion, nativity, and ethnic origins (all *p* < 0.004; Table 1). Within the International group, more men than women completed the survey compared to the two US-born cohorts. US White students had a higher self-reported health status than Multicultural and International students.

The plurality of students reported no religion, atheist, or agnostic (41.1%). Thirty-five percent of White students identified as Protestant. Slightly more Multicultural students were Catholic than Protestant. Among International students, the majority of religious adherents were Hindu or Buddhist (26.9%; *p* < 0.001). The International cohort tended to be older, in graduate school, non-native English speakers, and Asian Indian (30.9%), East Asian (29.1%), or MENA+ (12.4%). Multicultural students were 41.0% Latin American, followed by 21.8% East Asian and 15.7% African American or Black.

### 3.2. Influences on Food Choice and Meal Characteristics by Nativity–Ethnicity

Students ranked seven influences on food choice in the order of taste, price, convenience, nutrition, familiarity, healthfulness, and environmental sustainability [34]. International students classified convenience significantly lower than their peers (*p* = 0.019). Multicultural students ranked nutrition (*p* = 0.045) and healthfulness (*p* = 0.038) lower compared to both US White and International students. The other influences on food choice were not significantly different by nativity–ethnicity (Table 2).

On average, students ate homemade meals 3–4 days a week. Restaurant or take-out food, including campus dining, was the meal source 1–2 days a week. Fast-food meals were less frequent, at 1–3 days per month. Multicultural students had the most visits to a restaurant, take-out, campus dining, and fast-food outlets (all *p* < 0.001). International students had the lowest frequency of eating at fast-food businesses and the highest frequency of eating homemade meals and using free food options compared to others (*p* < 0.001). International students were the most confident in their ability to cook a meal. International students were the least likely to have a campus meal plan, and most likely to be the main food provider (both *p* < 0.001). A higher percentage of International students “rarely” ate meals with someone else (33%) and “always” ate meals with someone else (19.9%) compared to other groups (*p* < 0.001) (Table 2).

### 3.3. Dietary Fat and Hyper-Palatable Food Intakes by Nativity–Ethnicity

Over 50% of the students consumed 36% or more of their calories from fat, based on the FFQ [38,39]. White students had high or very high fat intakes (64%), followed by Multicultural (60%), and International students (49%) (*p* = 0.006). The consumption frequencies for the ‘red meat’ and ‘hyper-palatable foods’ subscales were significantly lower for International students than White or Multicultural cohorts (*p* < 0.001; Table 3).

Ordinal logistic regression (OLR; proportional odds model) modeled the 4-category dietary fat percentages of calories. A low percentage of calories from fat is the reference category. The simplest OLR model for all students showed significant positive influences of low-fat intake for being a non-native English speaker (OR 1.563; Wald *χ*2(1) = 8.879, *p* = 0.003) and older age (OR 1.045; Wald *χ*2(1) = 4.582, *p* = 0.032). Male gender (OR 0.588; Wald *χ*2(1) = 16.618, *p* < 0.001), on-campus housing (OR 0.613; Wald *χ*2(1) = 8.708, *p* = 0.003), and eating an evening meal with someone (OR 0.835; Wald *χ*2(1) = 15.664, *p* < 0.001) were negatively associated with low-fat intake. The goodness-of-fit deviance test showed that the model fit the observed data well, *χ*2(865) = 874.081; value/*df* = 1.010 [47]. Supplemental Table S1 provides the model details on the OLR analysis for all students, and for US White, Multicultural, and International students separately. Among Multicultural students, religious identity and eating evening meals with someone were negatively associated with low-fat intake, whereas being a native English speaker and being a woman were positively associated with low-fat intake. Among International students, low-fat intake was negatively associated with no campus meal plan and not being of Asian Indian ancestry. Higher self-reported health and being a woman were positively associated with low-fat intake.

### 3.4. Individual Food Frequency (FFQ) Item Comparisons by Nativity–Ethnicity

Analysis of variance (ANOVA) comparisons of the average consumption frequency of the individual dietary fat FFQ items showed significant differences for 13 of the 17 foods by nativity–ethnicity. Supplemental Table S2 displays the mean consumption frequencies by the three cohorts (all *p* ≤ 0.035). International students had higher intakes of eggs, whole milk, and fried chicken, and lower intakes of ground beef, cold cuts, bacon or sausage, French fries, pizza, cheese, and chips (all *p* ≤ 0.001) compared to White and Multicultural students. Post hoc analysis indicated that International students had lower frequencies of eating pastries and desserts than Multicultural (*p* = 0.020) and White (*p* = 0.032) students. International students used less margarine on foods compared to the White (*p* < 0.001) and Multicultural students (*p* = 0.015). Multicultural students had intermediate intakes of cold cuts (*p* = 0.048) compared to White and International students (*p* < 0.001). Cheese frequency was significantly different for Multicultural students compared to both White and International students (both *p* < 0.001).

### 3.5. Perceived Dietary Changes for Selected Foods by Nativity–Ethnicity

Table 4 displays frequency distributions for perceived change in dietary intakes for meats, hyper-palatable items, and starchy bread-type foods. Perceived changes in intakes were significantly different by nativity–ethnicity for all food groups (all *p* < 0.001). Over 10% of Multicultural and 13% of International students did not eat red meat. Increased red meat intakes were observed for International and Multicultural students, but decreased for White students (*p* < 0.001). Poultry, desserts, cheese and dairy foods, and bread intakes increased the most for International students. Snack food consumption increased significantly for Multicultural and International students compared to White students.

A lower percentage of White students abstained from alcohol, and a higher percentage increased alcohol use, compared to their peers (Table 5). Multicultural students increased their alcohol intake more than International students, but less than White students. About 50% of all respondents reported increased drinking of caffeinated beverages and hot drinks. Greater percentages of White and Multicultural students did not drink these at all. Energy drink usage increased the most for Multicultural students, whereas International students were less likely to consume them. Over 41% of International students reported an increase in carbonated beverage intake.

For nutrient-rich foods, International students increased their intake of fruits and berries, while Multicultural students reported that their consumption of these decreased (*p* = 0.030). Vegetable intakes stayed the same for the majority of students, with International students noting a decrease in comparison to their peers (*p* = 0.027). Over 18% of White and Multicultural students reported not eating any pulses. Multicultural students had a significant decrease, and International students had a significant increase in pulse intakes. White and Multicultural students were less likely to eat nuts and seeds compared to International students. Multicultural students reported the least increase in consumption of nuts and seeds (Table 6).

### 3.6. Food Attitudes Related to Diet Since Attending College

While most students agreed their diet had changed since college, higher percentages of Multicultural and International students strongly agreed this was true for them (*p* < 0.001). Multicultural and International students were significantly more likely to disagree that they ate the same foods all the time, compared to White students (*p* < 0.001). Multicultural and International groups had significantly greater access barriers to cultural foods, stating they often or very often had issues with desired foods being unavailable (*p* < 0.001; Table 7).

### 3.7. Ethnic Society Immersion (ESI) and Dominant Society Immersion (DSI) Scales

Thirty-eight percent (118/312) of US White students indicated their ancestral culture influenced food choices, and had significantly higher mean ESI and DSI scores than others. Cultures given were White only (38%), German only (11%), German with other European (36%), and other (15%). No one was a 2nd or 3rd generation descendent. ESI and DSI scores for US White students were not analyzed further.

Eighty-two percent (188/229) of the Multicultural students completed the SMAS. Their mean ESI score was significantly lower than that of the White and International cohorts. The mean Multicultural DSI score was intermediate between the means for White and International students. The International students exhibited the highest ESI scores and the lowest DSI scores (Table 8).

Among the four subgroups of the Multicultural students, post hoc comparisons showed US Latinos had significantly higher ESI scores than US East Asian (*p* = 0.001), and ‘other’ US ethnicity groups (comprising 16 Southeast Asians, 14 Asian Indians, and 8 Native American and other; *p* < 0.001). For the five International student subgroups, the mean ESI values were highest for the Asian Indian, East Asian, and MENA+ cohorts. The White European and the ‘other’ group (comprising 14 Africans, 11 Southeast Asians, and 15 Latin Americans) were 2–3 points lower than the other International groups (*p* = 0.043). For the DSI score, Multicultural and International students were significantly lower than US White students (all *p* < 0.001). One-way ANOVA main effects were not significant for the four Multicultural subgroups. The five International ethnic subgroups were significantly different from each other (*p* = 0.001). White Europeans had the highest mean DSI score, followed by Asian Indians. White European mean DSI scores were significantly higher than those of East Asian (*p* = 0.006) and MENA+ (*p* = 0.019) students. Almost 40% of International students had been in the US for 1 year or less. A larger percentage of MENA+ students were new arrivals (Table 8).

GLM analysis estimated the impact of theoretical predictors of the ESI and DSI components for the Multicultural and International student subgroups (Supplemental Table S3). For the Multicultural students, higher ESI scores were associated with being Latino and having a lack of cultural or ethnic foods (adjusted *R*^2^ = 0.099). The DSI model explained 11.9% of variance, with positive predictors including a greater cooking self-efficacy, being Latino, and having a lack of cultural or ethnic foods. For International students, the GLM explained 13% of the variance in ESI scores, with being religious, belief that one’s diet was not shifting to American foods, older age, and East Asian or Asian Indian origins all positive predictors. The DSI model explained almost 21% of the observed variance, with self-reported health, being a non-native English speaker, acknowledgement of diet shifting to American foods, lack of access to foods for religious purposes, not being of Asian Indian origin, and longer time in the US contributing significantly to model fit.

## 4. Results—Multicultural and International Ethnic Subgroups

### 4.1. Dietary Fat and Hyper-Palatable Food Intake by Multicultural and International Subgroups

For the Multicultural students, only bacon or breakfast sausage intakes were significantly different among the 17 FFQ items. Intakes were significantly higher for US East Asian and ‘other’ students compared to US Latinos and African American or Black students (*p* = 0.029). Comparisons of the percentage of calories from fat and red meat and hyper-palatable food scales were not significantly different among the four Multicultural ethnic subgroups. GLM analysis estimated the impact of various predictors of the red meat and hyper-palatable foods subscales for Multicultural students. For the red meat subscale, the model accounted for 23.8% of the variance. Being married, a man, and religious were positive predictors of red meat intake, whereas having a campus meal plan was negatively associated. For the hyper-palatable food subscale, the GLM only predicted 8% of the variance. Identifying as being East Asian, being an undergraduate, eating meals with someone, being a man, and being single were associated with higher hyper-palatable food intakes (Supplemental Table S4). Note that the ESI and DSI acculturation scores were not predictive of the Multicultural food subscale outcomes in this sample.

Table 9 shows the frequency distributions for eight FFQ items that were significantly different by International subgroups. Asian Indians ate significantly less red meat, cold cuts or lunch meat, ground meat, or bacon and sausage (all *p* < 0.001) than the others, and had the highest frequencies for whole milk intake. East Asians had high intakes of red meat and lower intake frequencies for pizza. The MENA+ subgroup had low intakes of cold cuts, bacon, or sausage, similar to those of Asian Indians, but had higher red meat intakes than the latter. Europeans had higher intakes of cheese, cold cuts, and the use of margarine, etc., on foods.

Supplemental Table S5 illustrates the frequency distributions for the percent calories from fat and the means of the red meat and hyper-palatable foods scales for the International subgroups. Only the red meat scale was significantly different, with Asian Indians having the lowest consumption (7.25 ± 3.6), followed by MENA+ (8.51 ± 2.9), Other (9.59 ± 2.8), East Asians (10.80 ± 3.5), and Europeans with the highest intake (11.27 ± 3.8; *p* < 0.001). The impact predictors of the food scales for International students were estimated by a GLM (Table 10). Prior to this GLM analysis, the ‘other’ ethnic category was excluded due to it being a small heterogeneous group. The model predicting red meat consumption accounted for 31.5% of the variance. Red meat intake was significantly associated with male gender, not being of Asian Indian or MENA+ origin, living on-campus, and lower ESI scores (all *p* < 0.004). The GLM predicting hyper-palatable food consumption explained 13.3% of the variance. Higher intake of these foods was associated with not being of East Asian origin, younger age, and poorer self-reported health (all *p* < 0.001).

### 4.2. Exploratory Questions on International Student Subgroup Intakes of Cultural Foods

Previous research at the university suggested that International students experience difficulty in finding cultural or ethnic foods, and that their diets changed while in the US [30]. International students were asked about their consumption patterns of eight signature foods for dietary acculturation (Table 11) [6,7,15,18,29,40,43]. There were no significant differences by cultural groups for intakes of fruits and berries or carbonated beverages. Fifty-nine percent reported eating fruits and berries three or more times per week, and 34% drank carbonated beverages one time per month or less. Post hoc analysis showed significant differences in consumption frequencies for rice, noodles, pulses, flatbreads, and soybeans. Asian Indian rice intakes were higher than for MENA+, European, and ‘other’ groups (*p* < 0.035), whereas noodle or pasta consumption was less than the others, except for MENA+ (*p* < 0.003). Pulses and flatbreads were consumed the most by Asian Indians (*p* < 0.004; *p* < 0.005, respectively). East Asian rice consumption frequency was similar to other groups except Europeans (*p* < 0.001). Noodle (*p* < 0.009) and pulse (*p* < 0.001) intakes were higher than for Asian Indians and MENA. Soups and stews were more frequent among East Asians than Asian Indians, MENA+, or Europeans (*p* < 0.011), and flatbreads were less common than for the other cohorts (*p* < 0.060). East Asian soybean intakes were greater than all other subgroups (*p* < 0.001). The MENA+ cohort ate less rice than Asian Indians, fewer noodles than East Asians, and more of both than European students. Pulse intakes were intermediate between Asian Indians and East Asians (*p* < 0.004). MENA+ students ate soups and stews less frequently than did East Asians but similar to the other groups (*p* = 0.010). MENA+ flatbread intakes were midway between Asian Indian and East Asian frequencies (*p* < 0.005). The two Asian groups had higher soybean intakes than other groups (*p* < 0.005).

Other exploratory measures included dietary habits of native food consumption and views on native food availability and healthfulness (Table 12). East Asians and MENA+ students had the highest frequency of eating native foods most days (*p* = 0.001). Asian Indian, East Asian, and European cohorts were most likely to report eating native foods at dinner, whereas MENA+ students were most likely to consume native foods at lunch or for nearly all meals. European students were most likely to agree that their diet was shifting towards American foods, and MENA+ students were most likely to state ‘maybe’ (*p* = 0.001). The majority of International students agreed their native diet was healthier. Asian Indian, East Asian, and MENA+ students were most likely to agree that native foods were ‘very often–often’ unavailable (*p* < 0.001).

## 5. Discussion

The study’s primary objectives were to compare meal characteristics, dietary intakes, and perceived changes since starting college among International, Multicultural, and US White students at a Midwest university in the US. The majority of International students were older, graduate students, practiced a religion that restricted meat or types of meat, and lived off-campus. They ate meals at home with other people and were in control of food acquisition. These individual characteristics suggest they were more self-reliant in meal provision and making food choices. While this group reported the greatest increases in red meats and hyper-palatable foods, overall consumption was still less than that of U.S.-born White and Multicultural peers. The White and Multicultural students were similar to each other on most meal characteristics, but the latter were more dependent on on-campus dining. Secondary objectives aimed to examine food consumption shifts by cultural group and acculturation differences within the Multicultural and International cohorts. Within this analysis, important heterogeneity emerged within the International subgroups. Asian Indian and MENA+ students consumed substantially less red meat and processed meats than East Asian and European peers, suggesting that religious practices, cultural food traditions, and food availability may shape dietary adaptation more strongly than acculturation alone. The study adds to public health nutrition research by documenting and comparing differences among International subculture groups and aids in the identification of those with greater nutritional risk.

US groups consumed higher percentages of calories from fat, and had higher scores on the subscales for red meats and high-fat, high-carbohydrate foods compared to International students. College students are noted for having high-fat dietary consumption [1,4,11]. Comparison of the three nativity–ethnicity cohorts indicates that net fat consumption percentages were lower among International students. However, when asked about their perceived dietary change, over 40% of International students reported increased intakes of red meats and hyper-palatable foods since college. While these perceived increases were of a lower magnitude than for the other two cohorts, this finding suggests a degree of dietary acculturation rather than full adaptation of the dominant culture. This observation is further supported by almost 70% of International students stating their diet is, or may be, shifting towards American patterns. Almohanna et al. reported similar findings in a smaller sample of heterogeneous cultures [7]. Increased intakes of calorie-dense, satiating hyper-palatable foods can lead to the replacement of nutrient-rich foods and possibly unintended weight gain [13,16].

The dramatic increase in alcoholic beverage intake, particularly binge drinking, among US college students is a prominent factor for risky behavior, including accidents. Schulenberg et al. note that college students were less likely to have already drunk alcohol in high school than were their non-college-bound peers [20]. While International students also reported increased use of alcohol, it was only half the increase in US White students. The majority of all respondents reported increases in hot beverages like coffee and tea, and nearly all International groups in the sample (Asian Indian, East Asian, MENA+) drank these. Energy drinks were consumed the most by Multicultural students, with minimal increase among their International counterparts. These beverages may appeal due to their high caffeine content, but are high in sugar and relatively expensive compared to other drinks. Furthermore, caffeinated beverages pose health risk factors such as reduced sleep and increased anxiety, and thus may be counter-productive to the perceived rationale of enhancing mental acuity [19]. International students had a marked increase in consumption of carbonated beverages or sodas. Like energy drinks, these are high in calories from sugar with variable levels of caffeine. Further inquiry into why or how these beverage changes occur would add to the understanding of these consumption shifts. While not inherently negative in small amounts, reliance on high-calorie beverages, including alcohol, can increase weight gain and displace more nutrient-dense beverages.

US White students increased their intake of pulses and leafy green vegetables more than did their peer groups. Exposure to new foods as part of the college experience may encourage consumption [49]. It is possible that the availability of nutrient-rich foods may have influenced shifts in their perceived intakes. Qualitative research on the reasons for changes and more details on food sources would help explain the observed phenomenon. International students had marked increases in consumption of fruits and berries, as well as nuts and seeds. However, the majority of older International students retained elements of traditional dietary patterns, therefore consuming more nutrient-rich foods and fewer high-fat and hyper-palatable options.

Many previous studies focus on the ‘non-White’ category as a monolith or on a single culture. In the current study, the Multicultural student subgroups (Latinos, East Asians, African Americans, other) did not exhibit many distinctive differences in the analysis of the dietary fat food frequency items. The observed homogeneity may be due to undergraduate status, limited ethnic food intakes prior to college, and a small sample size for African Americans and others. In contrast, disaggregated analysis of the five ethnic subgroups among International students revealed cultural nuances in food intake. Hinduism, Islam, and Judaism restrict meats, or certain types of meats, particularly pork [18]. Red meats were not consumed as frequently by Asian Indian, East Asian, and MENA+ International students who may practice one of these religions.

Another key objective of the research was to evaluate the influence of acculturation on food choice. Theoretically, dietary acculturation is one of the last changes prior to assimilation. Some changes may be positive for health, like replacing ghee or lard with a less saturated cooking oil. Most attention has been given to negative changes in the diet. A frequent assumption is that the traditional or native diet of most immigrants is healthier [50]. Increased dietary fat and hyper-palatable food intakes and declines in nutrient-rich foods are expected with acculturation. Yet, with the global nutrition transition, access to ultra-processed foods is already ubiquitous in most countries [16,21].

Although the ESI scores among Multicultural students were higher for Latino and African American students than those of East Asian ancestry, the evaluation of the linkage between acculturation and dietary parameters was not significant. Further investigation of these relationships is warranted. Among International students, ESI was very similar across the five cultural subgroups. On the other hand, the DSI was highest for European and Asian Indian students, and lowest for East Asian and MENA+ subgroups. English fluency and familiarity with US culture are likely drivers of these differences. Other studies suggest students from East Asian countries experience more stress from acculturation [12,51]. The magnitude of language and sociocultural differences can overwhelm and impede deeper immersion into the new country. English-speaking International students at a Polish university found similar concerns. When off campus, a lack of reading and speaking fluency in Polish led to social isolation and angst [52].

GLM estimation, which included the ESI and DSI acculturation scales, was used to predict red meat consumption scale scores. Analysis within the International cohort confirmed that greater ESI scores resulted in lower intakes, whereas DSI scores were not predictive. Gender played a notable role, in which men reported higher meat consumption, consistent with previous findings that men are more likely to adopt majority behaviors, as they may not have a background in cooking and preparing their own food [2]. Furthermore, students who did not identify as Asian Indian or MENA+ were more likely to consume red meat, reflecting sociocultural norms [18]. Lastly, those living on-campus were more likely to consume red meat, suggesting campus food environments or prevailing norms may influence red meat intake. While the GLM explained a modest amount of variance in red meat intake, linear modeling for the prediction of high-carbohydrate, high-fat intake explained a smaller, yet meaningful portion of variance. Consumers of these hyper-palatable foods were younger, not East Asian, and had lower self-reported health. These findings imply that younger International students may be at a greater risk of increasing intake of less nutrient-dense, hyper-palatable foods, highlighting a potential target for early dietary guidance and intervention.

The observed heterogeneity between the subgroups and the small sample size of some suggest that further longitudinal research on cultural group self-identification, social networks, and larger samples in the same environment would help to explain the observed differences in the acculturation scales, but not in the dietary metrics. Guendelman et al. have demonstrated that social perceptions and a desire to fit in may influence immigrant food choices—particularly when challenged as not being ‘American’ [48].

Most non-US dietary acculturation research with International students has been qualitative and conducted in other English-speaking countries such as Australia, Canada, and the United Kingdom. The scoping reviews by Shi [8] and Millar [53] support similar trends across regions as observed in the current research. Dietary acculturation forces appear to alter food intakes, but students initiate active preservation of traditional ways where possible [53]. Edwards [40] is one of the few studies that have compared International students by cultures of origin, or the local country populace, to allow for a clear separation of effects.

### Strengths and Limitations

Strengths include direct comparison of multiple cultural groups in the same environment using identical evaluation tools. Disaggregation of groups beyond nativity aids in understanding the dietary patterns of Multicultural and International subculture groups. Oversampling of underrepresented populations captured important nuances in dietary intake and perceived attitudes toward some foods [8]. The sample sizes are relatively large compared to previous studies. The specificity of findings with local comparisons and disaggregation of ethnic groups is important to define the nature of solutions.

The study has several limitations. Participants were not specifically asked about their familiarity with foods and consumption practices prior to starting college, nor were the US dietary patterns before university attendance measured. Thus, data reflect perceived changes in dietary intake by the respondents. While convenient, FFQs need further validation across cultural groups. Future research should validate these tools with multiple 24 h recalls, and/or target specific foods deemed markers of acculturation pre/post immigration [42]. While the use of a validated dietary fat FFQ is important, inclusion of a similar fruit and vegetable FFQ would have strengthened the description and comparison of culturally nuanced dietary patterns. The sample sizes of cultural subgroups were adequate for statistical analysis, but cultures are heterogeneous, and findings may not be representative. However, this study provides disaggregated data to illuminate group differences as called for in previous research [8,15,49].

Future studies with longitudinal samples, including metrics for academic performance, weight status, occupational status, financial aid use, income, and food security measures, will clarify confounding between acculturation processes or financial restrictions in terms of changes in foodways. Pre and post-testing over time to document changes within the same students would add veracity to the observed trends seen in this study. Some Multicultural and International students may have social network connections with other cultural members. Increased social support can foster retention of cultural foods and increase access [15,49,50,51,52,53].

## 6. Conclusions

Older International students were more likely to retain traditional dietary patterns, yet not without increases in some hyper-palatable foods upon arrival in the US. In contrast, the US-born non-Hispanic White and Multicultural students were most likely to consume alcohol, red meat, and high-fat and high-carbohydrate foods. Subgroup analyses revealed that culture and religious background strongly influenced red-meat intake among International students. In contrast, the Multicultural subgroups, likely due to greater homogeneity and smaller sample sizes, showed no statistically significant differences among subculture groups. These findings suggest that both cultural background and socio-demographic factors influence dietary change during college, further highlighting the need for targeted interventions in these populations to improve nutrition and health status.

Drawing from the study findings, one of the first steps universities could take would be to improve access to culturally and religiously appropriate foods. This lack of familiar options for almost 50% of International students can add psychosocial stress and even increase food insecurity [26]. A second step could be cooking workshops or personalized information about how to adapt traditional recipes using affordable local ingredients. Tailored subgroup-specific support for people new to the US would reduce vulnerability. Disaggregating outreach by developing resources grouped by region, religion, or acculturation stage, with extra orientation and ongoing support for first- and second-year students would be beneficial for academic success as well. The efforts go beyond International students and include first-generation and Multicultural students, too. Thus, these culturally sensitive and practical steps could benefit all students through a welcoming campus food environment.

## Figures and Tables

**Table 1:** Demographic and ethnic background characteristics by nativity–ethnicity among Midwest university students.

	Total *n* = 823	US White 38%; 312	Multicultural 28%; 229	International 34%; 282	*p*
AGE IN YEARS ($\bar{x}$ ± SD)	22.7 ± 3.9	21.3 ± 2.7 _a_	20.9 ± 2.9 _a_	25.6 ± 4.1 _b_	<0.001
	%				
GENDER					0.003
Man	44.0	41.7 _a_	37.6 _a_	51.8 _b_	
Woman	56.0	58.3 _a_	62.4 _a_	48.2 _b_	
SELF-REPORTED HEALTH					0.004
Poor–Fair	15.9	13.5 _a_	16.6 _a_	18.1 _a_	
Good	41.3	36.5 _a_	45.9 _a_	42.9 _ab_	
Very Good	33.8	40.7 _a_	32.3 _ab_	27.3 _b_	
Excellent	9.0	9.3 _ab_	5.2 _b_	11.7 _a_	
ACADEMIC LEVEL					<0.001
Undergraduate	63.4	83.3 _a_	83.0 _a_	25.5 _b_	
Graduate	36.6	16.7 _a_	17.0 _a_	74.5 _b_	
HOUSING LOCATION					
On-campus or with parents	27.6	31.7 _a_	39.3 _a_	13.5 _b_	<0.001
Off-campus	72.4	68.3 _a_	60.7 _a_	86.5 _b_	
MARITAL STATUS					
Single	83.7	87.8 _a_	86.9 _a_	76.6 _b_	<0.001
Married/Cohabitating	16.3	12.2 _a_	13.1 _a_	23.4 _b_	
NATIVE ENGLISH SPEAKER					<0.001
No	37.7	0 _a_	23.0 _b_	89.6 _c_	
Yes	62.3	100 _a_	77.0 _b_	10.4 _c_	
GENERATION					
1st	35.7	0_a_	5.2 _b_	100 _c_	<0.001
2nd	17.5	2.6 _a_	59.4 _b_	0 _c_	
3rd–4th	10.9	17.6 _a_	15.3 _a_	0 _b_	
5th	35.8	79.8 _a_	20.1 _b_	0 _c_	
RELIGION					<0.001
None, Atheist, or Agnostic	41.1	39.4 _a_	48.5 _b_	35.1 _a_	
Roman Catholic and Orthodox	20.8	25.2 _a_	23.3 _a_	14.0 _b_	
Muslim or Jewish	5.0	0.3 _a_	0.9 _a_	13.6 _b_	
Hindu or Buddhist	11.2	0 _a_	7.0 _b_	26.9 _c_	
Protestant Sects	22.5	35.2 _a_	20.3 _b_	10.4 _c_	
ANCESTRAL ORIGINS					<0.001
White—European	42.2	100	0.4	12.1	
East Asian	16.0	0	21.8	29.1	
Latin American	13.2	0	41.0	5.3	
Asian Indian	12.3	0	6.1	30.9	
African American—Black	6.6	0	15.7	6.4	
Middle Eastern–North African	4.3	0	0	12.4	
Southeast Asian	3.5	0	7.9	3.9	
Native American—Other	1.9	0	7.0	0	

Each subscript letter (_a–c_) denotes a subset of categories whose column percentages do not differ significantly from each other at the 0.05 level based on Bonferroni post hoc comparisons. Pairwise differences were assessed using adjusted standardized residuals from chi-square tests.

**Table 2:** Food practices and meal constraints among Midwest university students by nativity–ethnicity (%, *n*).

	Total *n* = 823	US White 38%; 312	Multicultural 28%; 229	International 34%; 282	*p*
INFLUENCES ON FOOD CHOICE ^1^			$\bar{x}$ ± SD		
Taste	3.30 ± 2.0	3.32 ± 2.0	3.22 ± 1.9	3.34 ± 2.1	n.s.
Price or cost	3.38 ± 2.0	3.26 ± 1.9	3.31 ± 2.1	3.58 ± 1.9	n.s.
Convenience	3.88 ± 1.9	3.69 ± 1.9	3.84 ± 1.9	4.12 ± 1.8	0.019
Nutrition	3.94 ± 1.6	3.97 ± 1.6	4.11 ± 1.5	3.76 ± 1.7	0.045
Familiarity	4.11 ± 1.9	4.29 ± 1.8	4.08 ± 1.9	3.93 ± 1.9	n.s.
Healthfulness	4.18 ± 1.7	4.08 ± 1.7	4.41 ± 1.5	4.08 ± 1.7	0.038
Environmental sustainability	5.15 ± 2.3	5.31 ± 2.3	4.95 ± 2.4	5.14 ± 2.3	n.s.
MEAL LOCATION FREQUENCY ^2^			$\bar{x}$ ± SD		
Homemade food or meals	3.83 ± 1.3	3.73 ± 1.3 _a_	3.59 ± 1.3 _a_	4.12 ± 1.1 _b_	<0.001
Restaurant, take-out, or campus	3.03 ± 1.2	3.05 ± 1.2 _a_	3.32 ± 1.2 _b_	2.79 ± 1.0 _c_	<0.001
Fast food, e.g., McDonald’s	2.21 ± 0.8	2.18 ± 0.8 _a_	2.39 ± 0.9 _b_	2.09 ± 0.8 _a_	<0.001
Free meals—charitable organizations	1.13 ± 0.4	1.08 ± 0.4 _a_	1.12 ± 0.4 _a_	1.20 ± 0.5 _b_	0.005
SELF-CONFIDENCE IN COOKING	3.37 ± 0.8	3.33 ± 0.8 _ab_	3.28 ± 0.9 _b_	3.47 ± 10.8 _a_	0.025
HAS COOKING FACILITIES	%				
Yes	94.3	95.2	91.3	95.7	n.s.
No	5.7	4.8	8.7	4.3	
CAMPUS MEAL PLAN					
Yes	30.8	34.9 _a_	44.1 _a_	15.4 _b_	<0.001
No	69.2	65.1 _a_	55.9 _a_	84.6 _b_	
MAIN FOOD PROVIDER STATUS					
Someone else	39.8	41.9 _ab_	45.4 _b_	33.5 _a_	0.14
Main food provider	60.2	58.1 _ab_	54.6 _b_	66.7 _a_	
EAT EVENING MEAL WITH SOMEONE					
Rarely (0–10%)	26.1	22.1 _a_	23.1 _a_	33.0 _b_	<0.001
Seldom (25%)	19.8	19.9 _a_	20.5 _a_	19.1 _a_	
Sometimes (50%)	15.8	17.0 _ab_	20.5 _ab_	10.6 _a_	
Most times (75%)	23.5	27.2 _a_	25.8 _ab_	17.4 _b_	
Always (100%)	14.8	13.8 _ab_	10.0 _b_	19.9 _a_	

Each subscript letter (_a–c_) denotes a subset of categories whose column percentages do not differ significantly from each other at the 0.05 level based on Bonferroni post hoc comparisons. Pairwise differences were assessed using adjusted standardized residuals from chi-square tests. n.s.= not significant. ^1^ Food choice rankings: 1: most influential, 7 = least influential; ^2^ meal location values: 1 = never, 2 = 1–3 days per month, 3 = 1–2 times per week, 4 = 3–4 days per week, 5 = almost every day [29].

**Table 3:** Percent of calories from fat and mean intakes of food frequency questionnaire subscale distributions by nativity–ethnicity among Midwest university students.

	Total *n* = 823	US White 38%; 312	Multicultural 28%; 229	International 34%; 282	*p*
PERCENT CALORIES FROM FAT		%			
Less than 30%	10.8	8.3 _a_	8.7 _ab_	15.2 _b_	
30–35% average	31.5	27.6 _a_	31.4 _a_	35.8 _a_	0.006
36–40% high	34.5	36.9 _a_	35.8 _a_	30.9 _a_	
40–50% very high	23.2	27.2 _a_	24.0 _ab_	18.1 _b_	
FFQ ITEM SUBSCALES		$\bar{x}$ ± SD			
Red meat and by-products	10.33 ± 3.7	11.03 ± 3.6 _a_	10.68 ± 3.5 _a_	9.27 ± 3.7 _b_	<0.001
High-fat and carbohydrate foods	12.00 ± 3.5	12.50 ± 3.3 _a_	12.46 ± 3.3 _a_	11.06 ± 3.6 _b_	<0.001

Each subscript letter (_a,b_) denotes a subset of categories whose column percentages do not differ significantly from each other at the 0.05 level based on Bonferroni post hoc comparisons. Pairwise differences were assessed using adjusted standardized residuals from chi-square tests.

**Table 4:** Self-reported consumption changes in meats, hyper-palatable, and starchy foods by Midwest university students by nativity–ethnicity.

	Total *n* = 823	US White 38%; 312	Multicultural 28%; 229	International 34%; 282	*p*
MEATS					
Beef, pork, lamb		%			<0.001
Does not eat	9.5	5.1 _a_	10.5 _ab_	13.5 _b_	
Decreased	30.0	40.1 _a_	31.0 _a_	18.1 _b_	
Stayed the same	40.0	46.2 _a_	38.9 _ab_	34.0 _b_	
Increased	20.5	8.7 _a_	19.7 _b_	34.4 _c_	
Poultry (chicken, turkey)					<0.001
Does not eat	6.8	5.1 _a_	6.6 _a_	8.9 _a_	
Decreased	18.3	22.4 _a_	21.0 _a_	11.7 _b_	
Stayed the same	39.4	43.3 _a_	39.7 _a_	34.8 _a_	
Increased	35.5	29.2 _a_	32.8 _a_	44.7 _b_	
HYPER-PALATABLE FOODS					
Desserts (cakes, sweets)					<0.001
Does not eat	3.0	1.6 _a_	4.4 _a_	3.6 _a_	
Decreased	23.1	26.3 _a_	23.2 _a_	19.6 _a_	
Stayed the same	34.2	40.7 _a_	32.9 _ab_	28.1 _b_	
Increased	39.6	31.4 _a_	39.5 _ab_	48.8 _b_	
Snack foods, chips					0.001
Does not eat	2.4	2.3 _ab_	0.9 _b_	3.9 _a_	
Decreased	20.2	24.6 _a_	20.6 _ab_	15.0 _b_	
Stayed the same	38.7	43.0 _a_	35.5 _a_	36.4 _a_	
Increased	38.7	30.1 _a_	43.0 _b_	44.6 _b_	
Cheese, yogurt, dairy foods					<0.001
Does not eat	3.5	3.0 _a_	4.8 _a_	2.9 _a_	
Decreased	17.4	16.8 _a_	20.2 _a_	15.8 _a_	
Stayed the same	46.4	54.6 _a_	45.6 _ab_	38.1 _b_	
Increased	32.7	25.7 _a_	29.4 _a_	43.2 _b_	
STARCHY FOODS					
Bread, flatbreads, dumplings					<0.001
Does not eat	2.2	1.6 _a_	0.9 _a_	3.9 _a_	
Decreased	14.9	12.3 _a_	19.1 _a_	14.3 _a_	
Stayed the same	49.9	62.1 _a_	48.4 _b_	37.5 _c_	
Increased	33.0	23.9 _a_	31.6 _a_	44.3 _b_	

Each subscript letter (_a–c_) denotes a subset of categories whose column percentages do not differ significantly from each other at the 0.05 level based on Bonferroni post hoc comparisons. Pairwise differences were assessed using adjusted standardized residuals from chi-square tests.

**Table 5:** Self-reported consumption changes in alcohol, caffeinated, and carbonated beverages of Midwest university students by nativity–ethnicity.

	Total *n* = 823	US White 38%; 312	Multicultural 28%; 229	International 34%; 282	*p*
ALCOHOLIC BEVERAGES		%			<0.001
Does not drink	31.4	23.5 _a_	37.6 _b_	35.1 _b_	
Decreased	9.1	7.2 _a_	6.6 _a_	13.1 _b_	
Stayed the same	18.9	16.3 _a_	14.2 _a_	25.5 _b_	
Increased	40.6	53.1 _a_	41.6 _b_	26.2 _c_	
COFFEE, TEA, HOT DRINKS					<0.001
Does not drink	14.2	20.6 _a_	14.0 _a_	7.2 _b_	
Decreased	9.1	5.8 _a_	9.2 _ab_	12.6 _b_	
Stayed the same	26.3	22.3 _a_	25.9 _ab_	31.0 _b_	
Increased	50.4	51.3 _a_	50.9 _a_	49.1 _a_	
ENERGY DRINKS					<0.001
Does not drink	44.6	44.9 _a_	39.0 _a_	48.8 _a_	
Decreased	11.0	9.6 _a_	10.1 _a_	13.2 _a_	
Stayed the same	20.1	19.2 _a_	17.5 _a_	23.1 _a_	
Increased	24.4	26.3 _a_	33.3 _a_	14.9 _b_	
CARBONATED BEVERAGES					0.001
Does not drink	16.4	17.4 _a_	19.3 _a_	13.1 _a_	
Decreased	22.2	28.0 _a_	21.5 _ab_	16.3 _b_	
Stayed the same	28.4	27.7 _a_	28.5 _a_	29.1 _a_	
Increased	33.0	27.0 _a_	30.7 _a_	41.5 _b_	

Each subscript letter (_a–c_) denotes a subset of categories whose column percentages do not differ significantly from each other at the 0.05 level based on Bonferroni post hoc comparisons. Pairwise differences were assessed using adjusted standardized residuals from chi-square tests.

**Table 6:** Self-reported consumption changes in nutrient-rich foods of Midwest university students by nativity–ethnicity.

	Total *n* = 823	US White 38%; 312	Multicultural 28%; 229	International 34%; 282	*p*
FRUITS AND BERRIES	%				0.030
Does not eat	1.7	1.3 _a_	1.8 _a_	2.1 _a_	
Decreased	30.0	29.6 _ab_	35.4 _a_	26.1 _a_	
Stayed the same	37.8	42.3 _a_	35.8 _a_	34.3 _a_	
Increased	30.5	26.7 _a_	27.0 _a_	37.5 _b_	
VEGETABLES, LEAFY GREENS					0.027
Does not eat	2.1	2.6 _a_	2.2 _a_	1.4 _a_	
Decreased	28.1	23.9 _a_	27.6 _ab_	33.3 _b_	
Stayed the same	38.3	36.2 _a_	37.8 _a_	40.9 _a_	
Increased	31.5	37.2 _a_	32.4 _ab_	24.3 _b_	
PULSES (BEANS, PEAS, LENTILS)					<0.001
Does not eat	15.2	18.3 _a_	18.8 _a_	8.9 _b_	
Decreased	21.9	16.4 _a_	19.2 _a_	30.1 _b_	
Stayed the same	37.6	33.8 _a_	38.9 _a_	40.8 _a_	
Increased	25.3	31.5 _a_	23.1 _b_	20.2 _b_	
NUTS AND SEEDS					0.001
Does not eat	10.9	14.7 _a_	13.7 _a_	4.3 _b_	
Decreased	19.5	16.3 _a_	21.2 _a_	21.6 _a_	
Stayed the same	47.3	48.1 _a_	46.0 _a_	47.5 _a_	
Increased	22.3	20.8 _a_	19.0 _a_	26.6 _a_	

Each subscript letter (_a,b_) denotes a subset of categories whose column percentages do not differ significantly from each other at the 0.05 level based on Bonferroni post hoc comparisons. Pairwise differences were assessed using adjusted standardized residuals from chi-square tests.

**Table 7:** Food attitudes and perceptions of Midwest university students by nativity–ethnicity.

	Total *n* = 823	US White 38%; 312	Multicultural 28%; 229	International 34%; 282	*p*
MY DIET HAS CHANGED AT COLLEGE	%				0.001
Strongly disagree	5.0	4.8 _a_	3.9 _a_	6.0 _a_	
Disagree	10.8	12.8 _a_	10.9 _a_	8.5 _a_	
Neutral	16.3	11.2 _a_	17.5 _ab_	20.9 _b_	
Agree	46.3	54.5 _a_	44.1 _ab_	39.0 _b_	
Strongly agree	21.6	16.7 _a_	23.6 _ab_	25.5 _b_	
I EAT THE SAME FOODS ALL THE TIME					<0.001
Strongly disagree	6.3	4.2 _a_	6.1 _a_	8.9 _a_	
Disagree	23.6	13.5 _a_	25.8 _b_	33.0 _b_	
Neutral	19.9	17.6 _a_	17.9 _a_	24.1 _a_	
Agree	35.2	44.2 _a_	36.2 _a_	24.5 _b_	
Strongly agree	14.9	20.5 _a_	14.0 _ab_	9.6 _b_	
CONSIDER MYSELF RELIGIOUS					<0.001
Strongly disagree	27.5	25.7 _a_	34.1 _b_	24.1 _a_	
Disagree	18.1	17.7 _a_	17.5 _a_	19.1 _a_	
Neutral	15.3	10.0 _a_	14.8 _ab_	21.6 _b_	
Agree	24.7	27.0 _a_	21.0 _a_	25.2 _a_	
Strongly agree	14.4	19.6 _a_	12.7 _ab_	9.9 _b_	
DIFFICULT FINDING CULTURAL FOODS					<0.001
Very often–often	28.6	4.8 _a_	34.9 _b_	49.8 _c_	
Sometimes	15.9	4.5 _a_	22.3 _b_	23.3 _b_	
Rarely never	55.6	90.7 _a_	42.8 _b_	26.9 _c_	

Each subscript letter (_a–c_) denotes a subset of categories whose column percentages do not differ significantly from each other at the 0.05 level based on Bonferroni post hoc comparisons. Pairwise differences were assessed using adjusted standardized residuals from chi-square tests.

**Table 8:** Age, Stephenson Multigroup Acculturation Scale (SMAS) mean distributions by nativity and ethnic subgroups and within Multicultural and International students.

ALL WITH SMAS	Total *n* = 588	US White 20%; 118	Multicultural 32%; 181		International 48%; 282	*p*
	⟵ $\bar{x}$ ± SD ⟶					
Age in years ($\bar{x}$ ± SD)	23.2 ± 4.2	21.3 ± 2.6 _a_	20.9 ± 2.9 _a_		25.6 ± 4.1 _b_	<0.001
Ethnic Society Immersion	54.7 ± 9.5	57.9 ± 10.8 _a_	51.1 ± 11.0 _b_		55.8 ± 6.8 _a_	<0.001
Dominant Society Immersion	46.5 ± 7.4	53.4 ± 2.5 _a_	49.1 ± 5.3 _b_		41.7 ± 6.7 _c_	<0.001
MULTICULTURAL	Latino 44%; 79	East Asian 23%; 42	African American 12%; 22		Other 21%; 38	
Age in years ($\bar{x}$ ± SD)	21.1 ± 3.0	20.9 ± 3.1	21.4 ± 3.1		20.1 ± 2.6	n.s.
Ethnic Society Immersion	54.9 ± 8.3 _a_	47.9 ± 12.9 _b_	51.9 ± 12.8 _a_		46.5 ± 10.0 _b_	<0.001
Dominant Society Immersion	48.4 ± 5.5	50.4 ± 4.9	48.0 ± 5.8		49.7 ± 5.1	n.s.
INTERNATIONAL	Asian Indian 31%; 87	East Asian 29%; 82	MENA+ 14%; 39	European 12%; 34	Other 15%; 40	*p*
Age in years ($\bar{x}$ ± SD)	25.5 ± 4.0	26.0 ± 4.1	27.0 ± 3.5	24.9 ± 4.6	24.9 ± 4.4	n.s.
Ethnic Society Immersion	56.7 ± 7.2	56.6 ± 5.8	56.0 ± 7.5	54.5 ± 5.6	53.2 ± 7.2	0.043
Dominant Society Immersion	42.9 ± 7.2 _ab_	40.0 ± 6.4 _a_	39.9 ± 7.0 _a_	44.7 ± 5.3 _b_	41.9 ± 6.0 _ab_	0.001
TIME IN THE US (total %)			%			0.016
1 year or less (39.6)	41.9 _ab_	29.6 _b_	61.1 _a_	40.0 _ab_	35.0 _ab_	
2–3 years (20.9)	23.3 _a_	18.5 _a_	16.7 _a_	13.3 _a_	30.0 _a_	
4–5 years (20.9)	18.6 _a_	27.2 _a_	13.9 _a_	33.3 _a_	10.0 _a_	
6–9 years (12.8)	9.3 _a_	21.0 _a_	2.8 _a_	13.3 _a_	12.5 _a_	
10+ years (5.9)	7.0 _a_	3.7 _a_	5.6 _a_	0 _a_	12.5 _a_	

Each subscript letter (_a–c_) denotes a subset of categories whose column percentages do not differ significantly from each other at the 0.05 level based on Bonferroni post hoc comparisons. Pairwise differences were assessed using adjusted standardized residuals from chi-square tests.

**Table 9:** Categorical intake frequencies from the dietary fat screener by International subgroups.

	Asian Indian 30.9%; 87	East Asian 29.1%; 82	MENA+ 13.8%; 39	European 11.7%; 34	Other 14.5%; 40	*p*
CHEESE OR CHEESE SPREADS	%					0.001
1 time per month or less	20.7 _a_	35.4 _a_	25.6 _a_	14.7 _a_	32.5 _a_	
2–3 times per month	23.0 _a_	29.3 _a_	15.4 _a_	8.8 _a_	22.5 _a_	
1–2 times per week	34.5 _a_	24.4 _a_	28.2 _a_	23.5 _a_	17.5 _a_	
3 or more times per week	21.8 _a_	11.0 _a_	30.8 _ab_	52.9 _b_	27.5 _ab_	
BEEF, PORK, STEAKS, ROAST, RIBS						<0.001
1 time per month or less	75.9 _a_	8.5 _b_	38.5 _c_	20.6 _bc_	30.0 _c_	
2–3 times per month	9.2 _a_	17.1 _a_	20.5 _a_	17.6 _a_	17.5 _a_	
1–2 times per week	10.3 _a_	32.9 _b_	25.6 _ab_	32.4 _b_	32.5 _b_	
3 or more times per week	4.6 _a_	41.5 _b_	15.4 _ac_	29.4 _bc_	20.0 _abc_	
WHOLE MILK (NOT LOW-FAT)						<0.001
1 time per month or less	25.3 _ab_	35.4 _ab_	12.8 _b_	50.0 _a_	47.5 _a_	
2–3 times per month	8.0 _a_	12.2 _a_	20.5 _a_	8.8 _a_	22.5 _a_	
1–2 times per week	11.5 _a_	12.2 _a_	25.6 _a_	8.8 _a_	15.0 _a_	
3 or more times per week	55.2 _a_	40.2 _ab_	41.0 _ab_	32.4 _ab_	15.0 _b_	
COLD CUTS, LUNCH MEAT, HAM						<0.001
1 time per month or less	78.2 _a_	45.1 _bc_	64.1 _ac_	20.6 _b_	47.5 _bc_	
2–3 times per month	8.0 _a_	29.3 _b_	20.5 _ab_	23.5 _ab_	30.0 _b_	
1–2 times per week	6.9 _a_	15.9 _ab_	12.8 _ab_	29.4 _b_	20.0 _ab_	
3 or more times per week	6.9 _a_	9.8 _ab_	2.6 _a_	26.5 _b_	2.5 _a_	
HAMBURGERS, GROUND BEEF						<0.001
1 time per month or less	58.6 _a_	26.8 _b_	38.5 _ab_	17.6 _b_	35.0 _ab_	
2–3 times per month	21.8 _a_	30.5 _a_	33.3 _a_	29.4 _a_	32.5 _a_	
1–2 times per week	9.2 _a_	35.4 _b_	17.9 _ab_	38.2 _b_	22.5 _ab_	
3 or more times per week	10.3 _a_	7.3 _a_	10.3 _a_	14.7 _a_	10.0 _a_	
MARGARINE, BUTTER, ON FOODS						0.023
1 time per month or less	17.2 _a_	31.7 _a_	20.5 _a_	29.4 _a_	27.5 _a_	
2–3 times per month	21.8 _a_	37.8 _a_	33.3 _a_	17.6 _a_	35.0 _a_	
1–2 times per week	33.3 _a_	17.1 _a_	30.8 _a_	20.6 _a_	25.0 _a_	
3 or more times per week	27.6 _ab_	13.4 _a_	15.4 _a_	33.3 _a_	12.2 _a_	
BACON OR BREAKFAST SAUSAGE						<0.001
1 time per month or less	81.6 _a_	48.8 _b_	82.1 _a_	50.0 _b_	52.5 _bc_	
2–3 times per month	3.4 _a_	26.8 _b_	7.7 _ab_	29.4 _b_	27.5 _b_	
1–2 times per week	10.3 _a_	17.1 _a_	5.1 _a_	11.8 _a_	12.5 _a_	
3 or more times per week	4.6 _a_	7.3 _a_	5.1 _a_	8.8 _a_	7.5 _a_	
PIZZA						0.013
1 time per month or less	28.7 _a_	41.5 _a_	17.9 _a_	23.5 _a_	35.0 _a_	
2–3 times per month	46.0 _a_	45.1 _a_	48.7 _a_	44.1 _a_	37.5 _a_	
1–2 times per week	18.4 _a_	12.2 _b_	30.8 _a_	29.4 _a_	12.5 _a_	
3 or more times per week	6.9 _ab_	1.2 _b_	2.6 _ab_	2.9 _ab_	15.0 _a_	

Percentages with differing subscripts within rows are significantly different at the *p* < 0.05 level, based on Bonferroni post hoc paired comparisons.

**Table 10:** Parameter estimates of a general linear model: measuring the strength of predictor variables of red meat and hyper-palatable food consumption subscale for International students (*n* = 242).

	Beta (*p* Value)	Partial Eta Squared	Observed Power	Adjusted *R*^2^
RED MEAT SUBSCALE				0.315
Gender (Man)	1.936 (<0.001)	0.085	0.996	
Not Asian Indian origin	3.958 (<0.001)	0.242	1.000	
Not MENA+ ^1^ origin	2.714 (<0.001)	0.082	0.996	
Lives on-campus	1.855 (0.004)	0.035	0.835	
Ethnic Society Immersion	−0.098 (0.002)	0.039	0.873	
HYPER-PALATABLE SUBSCALE				0.133
Not East Asian origins	1.524 (<0.001)	0.047	0.925	
Age in years	−0.257 (<0.001)	0.091	0.998	
Self-reported health	−0.549 (<0.001)	0.024	0.666	

^1^ MENA+ = Middle Eastern–North African–Other.

**Table 11:** Intake frequencies of eight cultural core foods by International subgroups (*n* = 267).

	Asian Indian 30.6%; 82	East Asian 29.9%; 79	MENA+ 13.8%; 37	European 12.3%; 33	Other 13.4%; 36	*p*
RICE	%					<0.001
1 time per month or less	0 _a_	5.1 _ab_	5.4 _ab_	17.6 _b_	5.7 _ab_	
2–3 times per month	2.4 _a_	6.3 _ab_	16.2 _bc_	20.6 _b_	14.3 _ab_	
1–2 times per week	22.0 _ab_	15.2 _a_	18.9 _a_	41.2 _a_	20.0 _ab_	
3 or more times per week	75.6 _a_	73.4 _a_	59.5 _a_	20.6 _b_	60.0 _a_	
NOODLES OR PASTA						<0.001
1 time per month or less	18.3 _a_	2.5 _b_	10.8 _ab_	2.9 _b_	8.6 _ab_	
2–3 times per month	36.6 _a_	17.5 _b_	29.7 _ab_	14.7 _b_	25.0 _ab_	
1–2 times per week	34.1 _a_	37.5 _a_	35.1 _a_	47.1 _a_	30.6 _a_	
3 or more times per week	11.0 _a_	42.5 _b_	24.3 _ab_	35.3 _b_	37.1 _b_	
PULSES						<0.001
1 time per month or less	6.1 _a_	37.5 _b_	16.2 _ab_	30.3 _b_	37.1 _b_	
2–3 times per month	17.1 _a_	33.8 _a_	18.9 _a_	30.3 _a_	17.1 _a_	
1–2 times per week	29.3 _a_	21.3 _a_	40.5 _a_	24.2 _a_	17.1 _a_	
3 or more times per week	47.6 _a_	7.5 _b_	24.3 _abc_	15.2 _bc_	28.6 _ac_	
SOUPS AND STEWS						0.009
1 time per month or less	30.9 _a_	11.3 _b_	35.1 _a_	26.5 _ab_	20.6 _ab_	
2–3 times per month	35.8 _a_	22.5 _a_	27.0 _a_	35.3 _a_	29.4 _a_	
1–2 times per week	13.6 _a_	38.8 _b_	18.9 _ab_	26.5 _ab_	23.5 _ab_	
3 or more times per week	19.8 _a_	27.5 _a_	18.9 _a_	11.8 _a_	26.5 _a_	
FLATBREADS						<0.001
1 time per month or less	9.0 _a_	46.3 _b_	25.0 _ab_	33.3 _b_	45.7 _b_	
2–3 times per month	11.5 _a_	32.5 _b_	19.4 _ab_	27.3 _ab_	25.7 _ab_	
1–2 times per week	34.6 _a_	15.0 _b_	25.0 _ab_	21.2 _ab_	17.1 _ab_	
3 or more times per week	44.9 _a_	6.3 _b_	30.6 _ac_	18.2 _abc_	11.4 _bc_	
SOYBEANS						<0.001
1 time per month or less	50.6 _a_	16.5 _b_	81.1 _c_	65.6 _ac_	68.6 _ac_	
2–3 times per month	26.6 _ab_	36.7 _b_	10.8 _a_	18.8 _ab_	11.4 _ab_	
1–2 times per week	16.5 _a_	29.1 _a_	8.1 _a_	12.5 _a_	14.3 _a_	
3 or more times per week	6.3 _a_	17.7 _a_	0 _a_	3.1 _a_	5.7 _a_	

Percentages with differing subscripts within rows are significantly different at the *p* < 0.05 level, based on Bonferroni post hoc paired comparisons.

**Table 12:** Native food consumption frequencies and dietary habits of International students.

	Total n = 268	Asian Indian 30.6%; 82	East Asian 29.9%; 80	MENA+ 13.8%; 37	European 12.3%; 33	All Others 13.4%; 36	*p*
NATIVE FOOD FREQUENCY							
Never-rarely	18.7	14.6 _ab_	6.3 _b_	24.3 _abc_	41.2 _c_	28.6 _ac_	0.001
Sometimes (1–2 times a week	22.5	19.5 _a_	24.1 _a_	16.2 _a_	29.4 _a_	25.7 _a_	
Most days (3–5 times a week)	30.7	29.3 _ab_	43.0 _b_	32.4 _ab_	11.8 _a_	22.9 _ab_	
Always (6–7 times a week)	28.1	36.6 _a_	26.6 _a_	27.0 _a_	17.6 _a_	22.9 _a_	
NATIVE FOODS AT MEALS							
Do not eat native foods	5.6	1.2 _a_	1.3 _a_	10.8 _ab_	11.8 _ab_	14.3 _b_	0.001
Breakfast	1.9	1.2 _a_	0 _a_	2.7 _a_	5.9 _a_	2.9 _a_	
Lunch	19.8	14.6 _a_	16.3 _a_	29.7 _a_	20.6 _a_	28.6 _a_	
Snacks	1.1	0 _a_	1.3 _a_	2.7 _a_	0 _a_	1 _a_	
Dinner	50.0	51.2 _ab_	66.3 _b_	24.3 _a_	50.0 _ab_	37.1 _a_	
Almost all of my meals	21.6	31.7 _a_	15.0 _a_	29.7 _a_	11.8 _a_	14.3 _a_	
DIET SHIFTED TO AMERICAN							
Yes	31.1	35.4 _ab_	27.5 _ab_	11.1 _b_	50.0 _a_	31.4 _ab_	0.023
Maybe	38.6	40.2 _bc_	35.0 _a_	58.3 _a_	26.5 _a_	34.3 _ac_	
No	30.3	24.4 _a_	37.5 _a_	30.6 _a_	23.5 _a_	34.3 _a_	
NATIVE OR US DIET HEALTHIER							
Native Diet Foods	79.5	78.0	81.3	73.0	90.9	75.0	n.s.
American Diet Foods	3.7	2.4	5.0	8.1	0	2.8	
Mix of both	6.0	7.3	2.5	8.1	0	13.9	
Do not know	10.8	12.2	11.3	10.8	9.1	8.3	
NATIVE FOODS UNAVAILABLE							
Very Often-Often	49.8	59.3 _ab_	40.0 _bc_	59.0 _ab_	17.6 _c_	67.5 _a_	<0.001
Sometimes	23.3	22.1 _a_	26.3 _a_	23.1 _a_	29.4 _a_	15.0 _a_	
Rarely Never	26.9	18.6 _a_	33.8 _ab_	17.9 _a_	52.9 _b_	17.5 _a_	

Percentages with differing subscripts within rows are significantly different at the *p* < 0.05 level, based on Bonferroni post hoc paired comparisons.

## Data Availability

The data are available upon request from the corresponding author due to safeguarding the privacy of disaggregated groups.

## Supplementary Materials

The following supporting information can be downloaded at: https://www.mdpi.com/article/10.3390/nu18142331/s1, Table S1. Ordinal logistic regression (OLR) model for predictors of low percentage of calories from fat intake overall, and among US-born White, US-born Multicultural, and International students; Table S2. Average consumption frequency of individual food items from the dietary fat screener that were significantly different by nativity-ethnicity of Midwest university students (%; *n* = 823) [38,39]; Table S3. Parameter estimates of a general linear model: measuring the strength of predictor variables for ESI and DSI acculturation subscales for Multicultural (*n* = 188) and International students (*n* = 282); Table S4. Parameter estimates of a general linear model: measuring the strength of predictor variables of red meat and hyper-palatable food consumption subscales for Multicultural students; Table S5. Percent calories from fat and hyper-palatable food subscale means by ethnic subgroups among International students at a Midwest university.
