# Supplementary material for: Comparison of Perceived Dietary Intakes Among International, Multicultural, and Non-Hispanic White College Students
Source: Nutrients. 2026 Jul 16;18(14):2331. doi: 10.3390/nu18142331 (PMC13415008; doi:10.3390/nu18142331)
Supplement: Supplementary file 1 [file nutrients-18-02331-s001.zip › nutrients-4225027-supplementary.pdf]

nutrients

MDPI

Supplemental Table S1. Ordinal logistic regression (OLR) model for predictors of low percentage of calories from fat intake overall, and among US-born White, US-born Multicultural, and International students.

			95% Confidence Interval for Odds Ratio		
	B (SE)	Sig.	Lower	Odds Ratio	Upper
ALL STUDENTS					
Very High Fat Intake (40-50%)	-1.003 (0.4817)	0.037	0.143	0.367	0.942
Quite High Fat Intake (36-40%)	0.624 (0.4800)	0.193	0.729	1.867	4.783
Average Fat Intake (30-35%)	2.527 (0.4911)	<0.001	4.781	12.518	32.776
Male gender	-0.530 (0.1301)	<0.001	0.456	0.588	0.759
Eats evening meal with someone	-0.181 (0.0457)	<0.001	0.763	0.835	0.913
Non-Native English speaker	0.446 (0.1498)	0.003	1.165	1.563	2.096
On-campus housing	-0.489 (0.1656)	0.003	0.443	0.613	0.849
Age	0.044 (0.0205)	0.032	1.004	1.045	1.088
US WHITE COHORT					
Very High Fat Intake (40-50%)	-1.256 (1.0384)	0.226	0.037	0.285	2.179
Quite High Fat Intake (36-40%)	-0.542 (1.0341)	0.600	0.227	1.719	13.049
Average Fat Intake (30-35%)	2.524 (1.0533)	0.017	1.583	12.476	98.309
Eats evening meal with someone	-0.317 (0.0793)	<0.001	0.624	0.728	0.851
Male gender	-0.697 (0.2179)	0.001	0.325	0.498	0.763
Consider myself religious	-0.197 (0.0711)	0.005	0.714	0.821	0.944
No campus meal plan	-0.646 (0.2559)	0.021	0.317	0.317	0.866
Age	0.089 (0.0444)	0.045	1.002	1.093	1.193
MULTICULTURAL COHORT					
Very High Fat Intake (40-50%)	-2.637 (0.423)1	<0.001	0.031	0.072	0.164
Quite High Fat Intake (36-40%)	-0.960 (0.3878)	0.013	0.179	0.383	0.819
Average Fat Intake (30-35%)	1.089 (0.4124)	0.008	1.324	2.970	6.666
Consider myself religious	-0.299 (0.859)	<0.001	0.627	0.741	0.877
Eats evening meal with someone	-0.203 (0.0931)	0.029	0.680	0.816	0.980
Non-Native English Speaker	0.552 (0.2909)	0.058	0.982	1.736	3.070
Male gender	-0.462 (0.2524)	0.067	0.384	0.630	1.033
INTERNATIONAL COHORT					
Very High Fat Intake (40-50%)	-1.616 (0.4537)	<0.001	0.082	0.199	0.484
Quite High Fat Intake (36-40%)	-0.096 (0.4420)	0.828	0.382	0.909	2.160
Average Fat Intake (30-35%)	1.735 (0.4560)	<0.001	2.319	5.669	13.856
Male gender	-0.658 (0.2251)	0.003	0.333	0.518	0.805
No campus meal plan	-0.759 (0.3051)	0.013	0.257	0.468	0.852
Not Asian Indian	-0.492 (0.2420)	0.042	0.380	0.611	0.982
Self-Reported Health Status	0.230 (0.1223)	0.061	0.990	1.258	1.599

Supplemental Table S2. Average consumption frequency[1] of individual food items from the dietary fat screener that were significantly different by nativity-ethnicity of Midwest university students (%; *n* = 823) [38, 39]

DIETARY FAT SCREENER ITEMS	Total	US White 37%; 312	Multicultural 28%; 229	International 35%; 282	***p***
Eggs (not Egg Beaters or egg whites)	3.11 ± 1.3	2.88 ± 1.2$_a$	2.98 ± 1.3$_a$	3.47 ± 1.3$_b$	<0.001
Cheese or cheese spreads (not low-fat)	3.00 ± 1.3	3.39 ± 1.2$_a$	2.98 ± 1.3$_b$	2.59 ± 1.3$_c$	<0.001
Corn chips, potato chips, popcorn, crackers	2.76 ± 1.1	2.96 ± 1.1$_a$	2.90 ± 1.1$_a$	2.44 ± 1.1$_b$	<0.001
Margarine, butter, or mayonnaise on foods	2.68 ± 1.2	2.84 ± 1.2$_a$	2.72 ± 1.1$_a$	2.46 ± 1.2$_b$	<0.001
French fries, fried potatoes	2.48 ± 1.0	2.55 ± 1.0$_a$	2.67 ± 1.0$_a$	2.26 ± 1.0$_b$	<0.001
Hamburgers, cheeseburgers, ground beef	2.50 ± 1.1	2.76 ± 1.1$_a$	2.68 ± 1.1$_a$	2.06 ± 1.0$_b$	<0.001
Doughnuts, pastries, cake, cookies	2.43 ± 1.1	2.49 ± 1.1$_a$	2.52 ± 1.1$_a$	2.30 ± 1.1$_b$	0.035
Whole milk (not low-fat or skim)	2.32 ± 1.5	2.02 ± 1.3$_a$	2.07 ± 1.4$_a$	2.84 ± 1.6$_b$	<0.001
Pizza	2.20 ± 0.9	2.35 ± 0.8$_a$	2.26 ± 1.0$_a$	1.99 ± 0.9$_b$	<0.001
Cold cuts, lunch meats, ham	2.15 ± 1.2	2.41 ± 1.2$_a$	2.22 ± 1.1$_b$	1.80 ± 1.1$_c$	<0.001
Salad dressings (not low-fat)	2.08 ± 1.1	2.29 ± 1.1$_a$	1.92 ± 1.0$_b$	1.98 ± 1.0$_b$	<0.001
Bacon or breakfast sausage	1.82 ± 1.0	1.93 ± 1.0$_a$	1.90 ± 1.0$_a$	1.62 ± 1.0$_b$	<0.001
Fried chicken	1.81 ± 1.0	1.60 ± 0.8$_a$	1.70 ± 0.9$_a$	2.09 ± 1.1$_b$	<0.001

[1] Food frequency response options: 1 = 1 time a month or less; 2 = 2-3 times a month; 3 = 1-2 times a week; 4 = 3=4 times a week; 5 = 5 times or more a week [35]. Same subscript letters (a-c) indicate column proportions that are not significantly different from each other. Pairwise differences were assessed using Bonferroni adjusted p-values in ANOVA.

Supplemental Table S3. Parameter estimates of a general linear model: measuring the strength of predictor variables for ESI and DSI acculturation subscales for Multicultural (*n*=188) and International students (*n*=282).

**Multicultural Students**	**Beta (*p* Value)**	**Partial Eta Squared**	**Observed Power**	**Adjusted R^2**
ETHNIC SOCIETY IMMERSION (ESI)				0.099
Latino origins	6.921 (<0.001)	0.096	0.993	
Lack of Cultural/ Ethnic Foods	1.287 (0.021)	0.028	0.637	
DOMINANT SOCIETY IMMERSION (DSI)				0.119
Lack of Cultural/ Ethnic Foods	1.066 (<0.001)	0.083	0.982	
Cooking Self-Efficacy	1.231 (0.003)	0.046	0.845	
Latino origins	0.930 (0.210)	0.009	0.240	

**International Students**	**Beta (*p* Value)**	**Partial Eta Squared**	**Observed Power**	**Adjusted R^2**
ETHNIC SOCIETY IMMERSION (ESI)				0.132
Considers self to be religious	1.284 (<0.001)	0.062	0.986	
Not Asian Indian origins	-2.258 (0.015)	0.091	0.964	
Not shifting to American diet	1.615 (0.001)	0.038	0.894	
Not East Asian origins	-2.824(0.004)	0.031	0.818	
Age in years	0.251 (0.012)	0.024	0.717	
DOMINANT SOCIETY IMMERSION (DSI)				0.209
Self-reported health	1.832 (<0.001)	0.073	0.993	
Native English speaker	-5.111 (<0.001)	0.062	0.982	
Shift to American diet	-1.710 (<0.001)	0.048	0.942	
Not Asian Indian origins	-2.752 (0.001)	0.042	0.908	
Religious foods unavailable	0.817 (0.004)	0.033	0.827	
Time in the US	0.788 (0.018)	0.022	0.660	

Supplemental Table S4. Parameter estimates of a general linear model: measuring the strength of predictor variables of red meat and hyper-palatable food consumption subscales for Multicultural students

	**Beta (*p* Value)**	**Partial Eta Squared**	**Observed Power**	**Adjusted R^2**
RED MEAT SUBSCALE				0.238
Gender (Man)	3.006 (<0.001)	0.170	1.000	
Campus Meal Plan	-1.180 (0.024)	0.030	0.560	
Marital Status (Single)	1.573 (0.050)	0.026	0.560	
Considers Self Religious	0.689 (<0.001)	0.091	0.964	
HYPER PALATABLE SUBSCALE				0.080
Undergraduate	1.481 (0.017)	0.033	0.672	
Married	-1.385 (0.063)	0.020	0.461	
Gender (Man)	0.991 (0.055)	0.021	0.485	
East Asian	1.501 (0.007)	0.040	0.768	
Eat meals with someone	0.412 (0.036)	0.025	0.557	

Supplemental Table S5. Percent calories from fat and hyper-palatable food subscale means by ethnic subgroups among International students at a Midwest university.

	**Asian Indian 30.9%; 87**	**East Asian 29.1%; 82**	**MENA+ 13.8%; 39**	**European 11.7%; 33**	**Other 14.5%; 41**	***p***
PERCENT CALORIES FROM FAT			%			
Less than 30%	19.5	14.6	12.8	9.1	14.6	
30–35% average	36.8	31.7	41.0	27.3	43.9	n.s.
36–40% high	27.6	35.4	25.6	39.4	26.8	
40–50% very high	16.1	18.3	20.5	24.2_{b}	14.6	
HYPER PALATABLE FOOD SCALES			$\bar{X} \pm$ SD			
Red meat and by-products	$7.25 \pm 3.6_{a}$	$10.80 \pm 3.5_{b}$	$8.51 \pm 2.9_{ac}$	$11.26 \pm 3.7_{bc}$	$9.55 \pm 2.9_{bc}$	<0.001
High fat & carbohydrate foods	11.48 ± 4.0	10.11 ± 3.1	11.59 ± 3.7	11.73 ± 2.9	11.05 ± 3.8	n.s.

Each subscript letter (a, b) denotes a subset of categories whose column percentages do not differ significantly from each other at the 0.05 level. Pairwise differences were assessed using adjusted standardized residuals from chi-square tests.
